# Vitamin D Supplementation on Carotid Remodeling and Stiffness in Obese Adolescents

**DOI:** 10.3390/nu14112296

**Published:** 2022-05-30

**Authors:** Christopher Morrissey, Marie-Josèphe Amiot, Aurelie Goncalves, Cecile Raverdy, Delphine Masson, Catherine Tardivel, Sandrine Gayrard, Myriam Carrère, Jean-Francois Landrier, Agnes Vinet, Antonia Perez-Martin

**Affiliations:** 1 LAPEC UPR4278—Laboratoire de Pharm-Ecologie Cardiovasculaire, Avignon University, 84029 Avignon, France; christopher.morrissey.france@gmail.com (C.M.); sandrine.gayrard@univ-avignon.fr (S.G.); 2 MOISA—Montpellier Interdisciplinary Center on Sustainable Agri-Food Systems, CIRAD—Centre de Coopération Internationale en Recherche Agronomique pour le Développement, Institut Agro-SupAgro, University Montpellier, INRAE—National Research Institute for Agriculture, Food and the Environment, CIHEAM-IAMM—International Centre for Advanced Mediterranean Agronomic Studies-Mediterranean Agronomic Institute of Montpellier, 34090 Montpellier, France; marie-josephe.amiot-carlin@inrae.fr (M.-J.A.); myriam.carrere@inrae.fr (M.C.); 3 APSY-V—Laboratoire APSY-v, University Nimes, 30021 Nîmes, France; aurelie.goncalves@unimes.fr; 4 SSR—Centre de Soins de Suite et de Réadaptation, Institut Saint Pierre, 34250 Palavas-les-Flots, France; raverdy.c@institut-st-pierre.fr (C.R.); masson.d@institut-st-pierre.fr (D.M.); 5 UMR C2VN—Center for CardioVascular and Nutrition Research, Aix Marseille University, INSERM—National Institute of Health and Medical Research, INRAE—National Research Institute for Agriculture, Food and the Environment, 13385 Marseille, France; c.tardivel@univ-amu.fr (C.T.); jean-francois.landrier@univ-amu.fr (J.-F.L.); 6 Vascular Medicine Laboratory, Nimes University Hospital, 30900 Nimes, France; antonia.perez.martin@chu-nimes.fr

**Keywords:** childhood obesity, vitamin D, arterial stiffness, intima-media thickness

## Abstract

Obesity is associated with vitamin D (VD) deficiency and arterial stiffness. This randomized control trial assessed the effects of VD supplementation during a weight-loss program on carotid intima-media thickness (IMT) and carotid compliance in obese adolescents. Participants were randomly assigned to receive either a 12-week lifestyle program with VD supplementation (*n* = 13), a lifestyle program without VD supplementation (*n* = 13) or a control group composed of normal-weight adolescents (*n* = 18). Serum total and free 25-hydroxyvitamin D (25(OH)D), IMT and carotid compliance were measured before and after the trial. Insufficiency in 25(OH)D concentration was found in 73% of obese participants compared to 22% among controls. Obese adolescents had lower free 25(OH)D and displayed higher IMT but lower carotid compliance than controls. Free 25(OH)D and IMT were negatively correlated in adolescents displaying VD insufficiency at baseline. After three months, total and free 25(OH)D increased in both groups. The changes of IMT and carotid compliance were similar between groups. The changes in IMT were correlated with the changes in total 25(OH)D in obese adolescents with VD insufficiency at baseline (*r* = −0.59, *p* = 0.03). While the lifestyle program with VD supplementation did not affect carotid compliance, IMT reduction was improved in obese adolescents.

## 1. Introduction

Childhood obesity is associated with a lower concentration of vitamin D (VD) [1,2] and with increased intima-media thickness (IMT) and greater arterial stiffness, two early markers of atherosclerosis [3]. Among healthy and overweight adults [4,5] and healthy adolescent females [6], VD insufficiency is associated with increased arterial stiffness. Two recent meta-analyses concluded that vitamin D supplementation alone does not improve arterial stiffness in adults [7,8] but can improve arterial stiffness among obese adolescents [9]. In contrast, weight loss reduces arterial stiffness [10] and IMT in adults [11] and can increase VD concentration among adults [12], children [13] and adolescents [14]. The synergistic influence of weight-loss program–induced improvements and VD supplementation on arterial compliance remain unexplored. Thus, this study aims to investigate if a lifestyle program mediated weight loss (including aerobic training and diet) and VD supplementation can improve arterial compliance and reduce IMT among obese adolescents. 

## 2. Materials and Methods

### 2.1. Study Population

Normal weight and obese adolescents (12–17 year) were recruited to participate in this study. Adolescents enrolled in a pediatric weight management clinic in the south of France who had a body mass index (BMI) z-score > 3 (defined as severe obesity) were invited to participate. Eighteen puberty-matched normal-weight volunteers were recruited from the community to serve as controls. In order to avoid seasonal variations, all adolescents were considered for inclusion between March–April 2015 and March–April 2016. Written informed consent was obtained from all adolescents and their parents. The study was approved by Ethics Committee CPP Sud-Mediterranee III (2015-000060-34) and performed in accordance with the principles outlined in the Declaration of Helsinki. The larger VIDADO study was registered in the ClinicalTrials.gov database (NCT02400151, last update posted 19 June 2017), and the detailed experimental design and population recruitment procedure have been previously reported [15].

**Figure 1:**
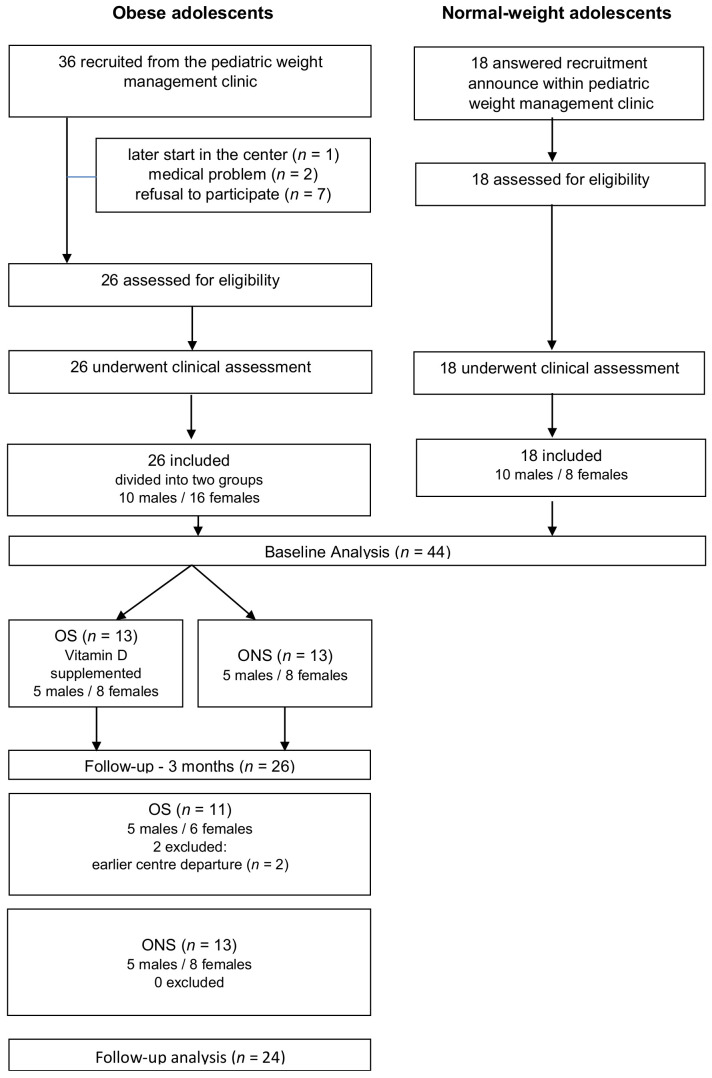
Flow chart of adolescent participants. OS: obese participants receiving vitamin D supplements; ONS: obese participants not receiving vitamin D supplements.

Obese adolescents were randomized by blocks of random size (2 or 4), with a 1:1 ratio into two groups (SAS software version 9.4, SAS Institute, Cary, NC, USA). All obese participants followed a three-month weight-loss program. As this was a double-blind trial, all participants were also asked to drink a fruit juice each morning with (OS, *n* = 13) or without (ONS; *n* = 13) VD supplementation (4000 IU/day of VD_3_ Uvedose^®^ (Crinex, Gentilly, France) 100,000 IU/2 mL) [16,17]. Two adolescents in the OS group left the clinic before the end of the program for personal reasons (at 3 months, OS = 11) (Figure 1). 

Throughout the clinic program, participants received both standard and personalized balanced meals prescribed by dieticians and based on a balanced distribution of macronutrients carbohydrates (55%), proteins (15%) and lipids (30% total, with less than 10% saturated fat). Total daily food intake was calculated to enable them to reach a negative balance of 500 kcal/day. Over three months of supervised moderate-to-vigorous intensity exercise, such as aerobic training consisting of 180 min/week, was required. Each session mostly involved aerobic exercise, including training circuit, boxing, basketball and Nordic walk with alternating short periods of high-intensity exercise interspersed among periods of lower intensity, as previously published [18].

At baseline and after three months, total and directly measured free 25-hydroxyvitamin D (25(OH)D) and additional inflammatory markers such as high-sensitive C-reactive protein (hsCRP) and interleukin 6 (IL-6) were measured in venous blood samples. Total 25(OH)D concentrations were determined by the electrochemiluminescence immunoassay (ECLIA) method, using Roche Diagnostics kits (Meylan, France) (Roche Elecsys vitamin D total). Free form of 25(OH)D was quantified by ELISA (DIAsource ImmunoAssays, Ottignies-Louvain-la-Neuve, Belgium). Hs-CRP and IL-6 were measured using commercially available ELISA kits purchased from R&D systems (Minneapolis, MN, USA) according to the manufacturer’s specifications. All vascular measurements were performed after overnight fasting in a quiet room with a controlled temperature between 22 and 24 °C using high-resolution vascular ultrasonography (MyLab30, Esaote SpA, Firenze, Italy), performed by the same observer (AV) blinded to group allocation. For the examination of the common carotid artery (CCA), the transducer was placed 2–3 cm proximal to the carotid bifurcation on the right side of the neck without compromising the internal jugular vein. Diastolic and systolic diameters were defined by the distance from the leading edge of the intima-lumen interface of the near wall and the lumen-intima interface of the far wall. The carotid IMT (mm), defined as the distance from the leading edge of the lumen-intima interface to the leading edge of the media-adventitia interface of the far wall, was measured and averaged automatically by the analysis software (MyLabDesk, Esaote, Italy) according to the Mannheim consensus. Carotid artery compliance (CAC, mm^2^/mmHg) was calculated as CAC = π(Ds2−Dd2)/4PP (Ds and Dd are systolic and diastolic arterial diameters, respectively; PP is pulse pressure, measured on the left arm by an automated system (Dinamap, GE Medical Systems, Milwaukee, WI, USA)). Within-subject variation was 1.8% for arterial diameters [19].

### 2.2. Statistical Method

Statistical analysis was performed with free R software (R Core Team, Vienna, Austria). The primary outcome variable was IMT. The sample size was calculated by considering a type I error rate of 5% (2-tailed) and a power of 85% based on Montero et al.’s findings on obese adolescents [20]. Calculations indicated that a sample size of 12 participants was required in each group to achieve statistical power. Due to the small sample size (<30), we performed non-parametric statistical tests involving median comparisons. We conducted a Mann–Whitney test between obese and normal-weight adolescents to establish a baseline. We also conducted Mann–Whitney tests to compare lifestyle only vs. lifestyle and VD supplementation. Then we computed Wilcoxon signed-rank tests to compare the effect of the program on all obese. Finally, Spearman correlations were used to explore relationships between total and free 25(OH)D and vascular outcomes. For follow-up analysis, a subgroup analysis with only the 18 obese adolescents deficient in vitamin D at baseline was also performed. Multiple stepwise linear regression analyses were conducted to identify the strongest predictors of delta IMT.

## 3. Results

Participant characteristics at baseline are described in Table 1. 

At baseline, obese participants in both groups had lower total 25(OH)D and lower free 25(OH)D (only measured in 21 obese participants) concentrations and higher CRP and IL-6 levels than normal-weight adolescents (NW). VD insufficiency (defined by 25(OH)D levels < 50 nmol/L [18]) was detected in 73% (18/26) of obese and 27% (5/18) normal-weight participants, respectively. CCA diameter and CCA-IMT were higher, and CCA compliance was lower in obese participants (Table 1). 

Concentrations in total and free 25(OH)D were not correlated with any CCA data. However, when adolescents with VD insufficiency at baseline (<50 nmol/L) were analysed, the correlation between free 25(OH)D and IMT became significant (*r* = −0.41, *p* = 0.04). In addition, total and free 25(OH)D concentrations were negatively correlated with inflammatory markers (IL-6 and hsCRP).

After the three-month interventional program, BMI, fat mass and CRP decreased. Total 25(OH)D increased in OS and ONS without any difference between both groups. Systolic and diastolic blood pressure did not change. All OS and only 60% ONS (8/13) reached a sufficient status in VD. Free 25(OH)D also increased in both groups, but contrary to baseline, free 25(OH)D became significantly higher in OS (*n* = 8) than in ONS (*n* = 9). Obese participants had significant CCA-IMT reduction, while CCA compliance increased in groups (Table 2). However, these changes were not significant in OS and ONS.

**Figure 2:**
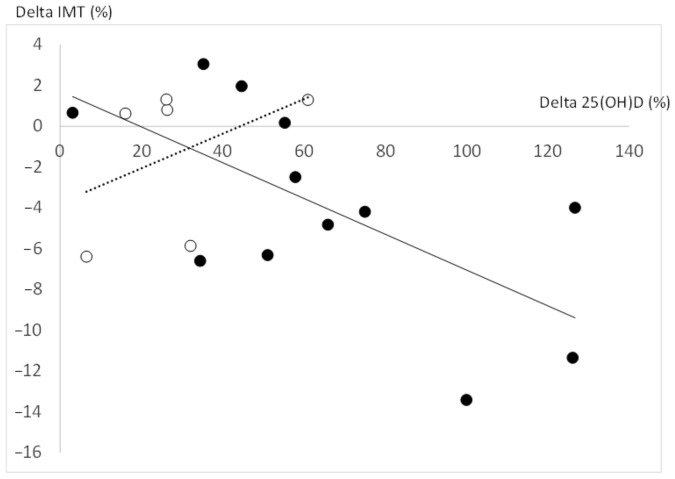
Correlations delta total 25(OH)D and delta IMT in all obese adolescents: obese participants with VD insufficiency at baseline (black circle) and obese participants without VD insufficiency at baseline (white circle). 25(OH)D: 25-hydroxyvitamin D; IMT: Intima-media thickness; VD: Vitamin D.

Delta change of each parameter did not differ between OS and ONS. Delta total and free 25(OH)D concentrations (%) were neither correlated with delta CCA compliance nor delta inflammatory markers. Delta total 25(OH)D concentrations (%) was only negatively correlated (at borderline) with delta IMT (%) (*r* = −0.43, *p* = 0.06). However, when we took into account only obese adolescents with VD insufficiency at baseline (<50 nmol/L), the correlation between delta total 25(OH)D and delta IMT became significant (*r* = −0.59, *p* = 0.03) (Figure 2).

Multivariate linear regression analyses were used to examine the relationships between delta IMT and age, changes in total 25(OH)D, fat mass, blood pressure and CRP. Only delta total 25(OH)D) emerged as an independent predictor of delta IMT, explaining between 25 and 37% of its variance according to the input variables used. 

## 4. Discussion

The main finding of this double-blind, randomized controlled trial was that improvement in 25(OH)D level was correlated with IMT reduction during a weight loss program in VD-insufficient obese adolescents. As IMT is a well-established surrogate marker of atherosclerosis and predictive of cardiovascular morbidity and mortality in adults [11], this result may add new evidence for a vasculoprotective function of VD and consequently may be an important pediatric health standpoint. Nevertheless, VD supplementation combined with diet and aerobic training did not enhance CCA compliance improvement in obese adolescents.

Similarly observed in other studies [1,2], 73% of obese participants had VD insufficiency (total 25(OH)D levels < 50 nmol/L). This VD status in obese adolescents may not be due to differences in vitamin D-protein binding as free 25(OH)D was also lower in obese participants than in controls. However, Miraglia et al. [21] reported a low total 25(OH)D but similar calculated free 25(OH)D in 10-year-old obese children compared to normal-weight ones. As suggested by Sollid et al. [22], calculations appear to overestimate the free 25(OH)D concentrations compared to the direct measurements, which may explain these different results. The reason for the low 25(OH)D observed among obese adolescents is not straightforward. Plausible explanations include limited skin exposure to ultraviolet (UV) B radiation due to less outdoor activity, the impaired ability of their skin to convert 7-dehydrocholesterol to VD_3_ and the sequestration of VD in adipose tissue and its volumetric dilution [23]. Whereas VD insufficiency is associated with increased arterial stiffness in adults [4,5], we did not observe any relationships between total 25(OH)D with arterial stiffness and IMT in obese adolescents. These results are not universal in obese children [24,25] and adolescents [6]. However, free 25(OH)D was significantly associated with IMT in adolescents with VD insufficiency at baseline. Beyond obesity, low 25(OH)D levels in childhood are associated with high-risk carotid IMT in adulthood [26]. 

The weight-loss program improved total and free 25(OH)D levels in all obese participants, presumably due to demobilization of the vitamin from the adipose tissue into the bloodstream and sun exposure. We observed an increase in total and free 25(OH)D in normal-weight adolescents during the same sunny period (+20% and +27%, respectively, data not shown). Similarly, Tzotzas et al. [27] reported that a 10% reduction in weight resulted in 34% increased VD concentration in VD-insufficient obese individuals at baseline. In the present study, obese participants lost weight (~8% of total body weight, which could translate into an increase in VD concentration of 27%. Although ONS was able to increase total 25(OH)D levels significantly (+35%), the VD status remained at the level of insufficiency in 40% ONS. In contrast, supplementation at 4000 IU/day for three months during a weight-loss program was effective in reaching adequate 25(OH)D levels. As VD insufficiency, higher IMT and lower CCA compliance coexisted in the obese participants, our combined program (diet, exercise plus VD supplementation) may represent an adequate time period to reverse these health complications.

The weight-loss program appeared to counterbalance the progression of CCA compliance with no additional effect of VD supplementation. Note that VD supplementation combined with the weight-loss program had no undesirable effect on CCA compliance. This result is in agreement with the conclusions of two recent meta-analyses [7,8] on the effect of VD supplementation on arterial stiffness. Our result added new knowledge on VD supplementation that, combined with a weight-loss program, did not have an enhanced or synergistic effect on carotid compliance. However, the weight-loss program resulted in a significant reduction in IMT among all obese participants, with the greatest IMT reduction achieved among obese adolescents who had VD insufficiency at baseline and better improved their total 25(OH)D status. Furthermore, delta 25(OH)D emerged from multivariate analysis as the only contributor of delta IMT by explaining 25% to 39% of the variance according to the input variables used. Collectively, these results may suggest a vasoprotective VD effect of VD supplementation and weight loss for this population. This result is similarly described in a recent meta-analysis of two randomized controlled studies, which reported that supplementation in VD significantly reduced IMT among adults [28]. Considering the role of inflammation in atherosclerosis [29], we can speculate that lowering CRP by VD, combined with weight reduction, might have beneficial anti-atherogenic effects. However, the lack of relationship between delta CRP changes and delta IMT weaken this hypothesis and merit further exploration.

The present study has several limitations. First, the relatively small sample size restricts the possibility of extrapolation to a wider population, despite statistical significance. Second, the outdoor activity time of participants was not collected. Third, it remains unclear which interventional factor causes the observed results because diet, exercise and VD supplementation may have a distinct or synergic effect on 25(OH)D levels and vascular profiles.

## 5. Conclusions

In conclusion, improving vitamin D status by supplementation during a weight loss program can help with IMT reduction. However, carotid compliance was not increased, and further longer programs should be explored. From a pediatric health standpoint, such effective programs that counteract vascular impairment in obese adolescents must be encouraged, especially among those with VD insufficiency. Further studies with more subjects and longer follow-up periods are needed to confirm the vasoprotective effect of VD and better understand the mechanisms underlying this effect.

## Figures and Tables

**Table 1:** Baseline characteristics of the study groups.

	Obese	Obese Subgroups		*p*-Value	Normal-Weight	*p*-Value
Characteristics	(*n* = 26)	OS (*n* = 13)	ONS (*n* = 13)	OS vs. ONS	(*n* = 18)	All OA vs. NW
Age (year)	14.2 (13.6–14.8)	14.6 (13.6–14.8)	14 (13.1–16.7)	0.959	14.8 (13.3–15.3)	0.535
Boys/girls (*n*)	10–16	5–8	5–8	1.0	10–8	0.728
BMI (kg/m^2^)	33.5 (31.2–36.2)	33.8 (31.3–36.5)	32.7 (31.1–36.3)	0.614	19.2 (18.5–20.2)	<0.0001
BMI Z-score	4.0 (3.5–4.5)	3.9 (3.4–4.7)	4.0 (3.4–4.6)	0.959	0.0 (−0.3–0.7)	<0.0001
Fat mass (%)	41.4 (39.8–43.2)	41.9 (39.8–43.6)	41.1 (36.5–44.3)	0.555	23.2 (18.4–27.7)	<0.0001
SBP (mmHg)	112 (106–120)	112 (106–123)	113 (106–120.5)	0.797	104 (100–108)	<0.001
DBP (mmHg)	66 (62–68)	59 (57–60)	61 (59–68)	0.039	60 (59–62)	0.004
Total 25(OH)D (nmol/L)	38 (35.4–47.8)	39 (38–55.8)	34.5 (32–58.6)	0.191	57 (48.1–67.7)	0.002
*n* deficiency in 25(OH)D	8	1	7	-	0	-
*n* insuficiency in 25(OH)D	18	9	9	-	5	-
Free 25(OH)D (pg/mL)	4.1 (1.9–5.3)	4.1 (1.9–5.3)	4.1 (2.4–4.8)	0.918	4.8 (2.6–6.2)	0.006
CRP (mg/mL)	6.1 (3.3–10.1)	7.9 (3.6–13.7)	4.4 (2.3–10.1)	0.331	0.3 (0.2–0.7)	<0.0001
IL-6 (pg/mL)	2.6 (1.7–2.7)	2.7 (1.8–2.9)	2.3 (1.6–2.7)	0.283	0.55 (0.5–1.1)	<0.0001
CCA diameter (mm)	5.73 (5.59–581)	5.77 (5.53–5.87)	5.70 (5.31–5.82)	0.355	5.05 (4.84–5.24)	<0.0001
CCA compliance (mm^2^/mmHg)	0.11 (0.09–0.12)	0.10 (0.06–0.16)	0.11 (0.08–0.12)	0.923	0.16 (0.12–0.18)	0.0017
CCA-IMT (mm)	0.64 (0.63–0.67)	0.63 (0.62–0.66)	0.66 (0.62–0.68)	0.342	0.61 (0.59–0.63)	0.0186

OA: Obese adolescents; OS: obese participants receiving vitamin D supplements; ONS: obese participants not receiving vitamin D supplements; NW: Normal-weight adolescents; BMI: Body mass index; SBP and DBP: Systolic and diastolic blood pressure; 25(OH)D: 25-hydroxyvitamin D; CRP: high-sensitive C-reactive protein; IL-6: Interleukin 6; CCA: Common carotid artery, IMT: Intima-media thickness.

**Table 2:** Changes in biological and vascular outcomes at three months.

	All Obese		*p*-Value	Changes from Baseline (in %)		*p*-Value
Outcome Variables	Baseline	3 Months		OS	ONS	
Total 25(OH)D (mmol/L)	38.0 (35.4–47.8)	67.0 (57.0–71.6)	<0.0001	55.2 (38.1–72.0)	34.9 (8.1–70.9)	0.132
Free 25(OH)D (mmol/L)	4.1 (1.9–5.3)	5.9 (4.1–8.3)	0.0001	59.4 (36.3–77.4)	27.4 (8.6–60.6)	0.336
BMI (kg/m^2^)	33.5 (31.2–36.2)	30.3 (29.6–32.5)	<0.0001	−8.5 (−11.2–−6.3)	−7.8 (−10.9–−5.6)	0.649
CRP (mg/mL)	6.1 (3.3–10.1)	2.0 (0.9–4.5)	0.0002	−48.8 (−81.6–−0.35)	−65 (−81.1–48)	0.331
IL-6 (pg/mL)	2.6 (1.7–2.7)	1.6 (1.1–1.9)	0.007	−36.9 (−76.9–1.7)	−36.3 (−60.3–−12.8)	1
Carotid diameters (mm)	5.73 (5.59–5.81)	5.66 (5.46–5.83)	0.678	0 (−1.1–2.5)	−1.5 (−4.4–3.5)	0.434
Carotid compliance (mm^2^/mmHg)	0.11 (0.09–0.12)	0.12 (0.10–0.15)	0.05	2.35 (−9.8–18.2)	15.5 (−5.3–58.1)	0.305
Intima-media thickness (mm)	0.64 (0.63–0.67)	0.63 (0.62–0.67)	0.034	−3.25 (−7.3–0.9)	0.61 (−6.4–2.1)	0.599

OS: obese participants receiving vitamin D supplements; ONS: obese participants not receiving vitamin D supplements; 25(OH)D: 25-hydroxyvitamin D; BMI: Body mass index; CRP: high-sensitive C-reactive protein; IL-6: Interleukin 6.

## Data Availability

The data presented in this study are available on request from the corresponding author.
